# Radioguided breast surgery for occult lesion localization – correlation between two methods

**DOI:** 10.1186/1756-9966-27-29

**Published:** 2008-08-15

**Authors:** Marcelo Moreno, Janete Eunice Wiltgen, Benito Bodanese, Ricardo Ludwig Schmitt, Bianca Gutfilen, Lea Mirian Barbosa da Fonseca

**Affiliations:** 1 Department of Surgery, Medicine School, Hospital Regional do Oeste, Universidade Comunitária Regional de Chapeco, Santa Catarina, Brazil; 2 Department of Radiology, Radimagem Clinic, Santa Catarina, Brazil; 3 Department of Oncology, Medicine School, Hospital Regional do Oeste, Chapeco, Brazil; 4 Department of Epidemiology, Medicine School, Universidade Comunitária Regional de Chapeco, Santa Catarina, Brazil; 5 Department of Radiology, Medicine School, Universidade Federal do Rio de Janeiro, Rio de Janeiro, Brazil

## Abstract

**Background:**

The detection of sub-clinical breast lesions has increased with screening mammography. Biopsy techniques can offer precision and agility in its execution, as well as patient comfort. This trial compares radioguided occult lesion localization (ROLL) and wire-guided localization (WL) of breast lesions. We investigate if a procedure at the ambulatorial level (ROLL) could lead to a better aesthetic result and less postoperative pain. In addition, we intend to demonstrate the efficacy of radioguided localization and removal of occult breast lesions using radiopharmaceuticals injected directly into the lesions and correlate radiological and histopathological findings.

**Methods:**

One hundred and twenty patients were randomized into two groups (59 WL and 61 ROLL). The patients were requested to score the cosmetic appearance of their breast after surgery, and a numerical rating scale was used to measure pain on the first postoperative day. Clearance margins were considered at ≥ 10 mm for invasive cancer, ≥ 5 mm for ductal carcinoma *in situ*, and ≥ 1 mm for benign disease. Patients were subsequently treated according to the definitive histological result. When appropriate, different statistical tests were used in order to test the significance between the two groups, considering a P value < 0.05 as statistically significant.

**Results:**

WL and ROLL located all the occult breast lesions successfully. In the ROLL group, the specimen volume was smaller and there were more cases with clear margins (P < 0.05). There were significant differences in mean time of hospital stay between WL and ROLL (21.42 vs. 2.56 hours), but not in operative time (39.4 vs. 29.9 minutes). There were significant differences in the subjective ease of the procedures as rated by the patients (cosmetic outcomes and postoperative pain).

**Conclusion:**

ROLL is an effective method for the excision of non-palpable breast lesions. It enables more careful planning of the cutaneous incision, leading to better aesthetic results, less postoperative symptoms, and smaller volumes of excised tissue.

## Background

The diagnosis of breast cancer has changed over the last years. Previously, about 50 to 70% of breast cancers were diagnosed through physical examination [1]. The detection of subclinical lesions has increased with screening mammography [2]. Thus, the need arose to develop minimally invasive techniques of locating and histological confirming small alterations [3,4]. Wire localization (WL) is a well-known technique in breast surgery where a malleable needle with a spear at its distal extremity is used to locate a lesion. Under mammography or ultrasound visualization, a needle is placed directly into an area suspicious as per the nature of the lesion [3,4]. There is a risk of needle displacement during the period between its positioning and retreat, mainly in breasts with a predominant fatty component [2,4]. This can represent an important complication in patients with mammary prosthesis, for example. In dense breasts, difficulty in positioning the needle localization device can occur. Cases of transected needles, pneumothorax, and other accidents have been described [2,5]. Needle localization of occult lesions is usually done under general anesthesia due to patient discomfort when the needle localization device is manipulated [5-7].

Radioguided occult lesion localization (ROLL) is a method that has been used since 1996 [8]. It was developed at the European Institute of Oncology in Milan, and is currently the standard of care in many breast surgery services. In this procedure, a radioactive labeling substance is used at the suspect site (under ultrasound or mammography guidance). The gamma-detecting probe guides the localization of a suspicious opacity or microcalcification cluster during the surgical procedure [6,9]. The cutaneous incision can be planned with better aesthetic results. In this method, a spear is not used; instead, a small portion of liquid makes the process less traumatic for patients. Local anesthesias for ROLL and patients' opinions as to the pain and postoperative aesthetic results have not been previously studied for effectiveness and patient acceptability [7,9].

The goal of this paper is to show the feasibility of performing the ROLL technique in an ambulatory setting, with shorter operative time and less patient morbidity, through careful surgical planning and the extraction of a smaller mammary sample. Therefore, these advantages make it the preferred method for occult breast lesion localization with diagnostic intention.

## Methods

**Figure 1:**
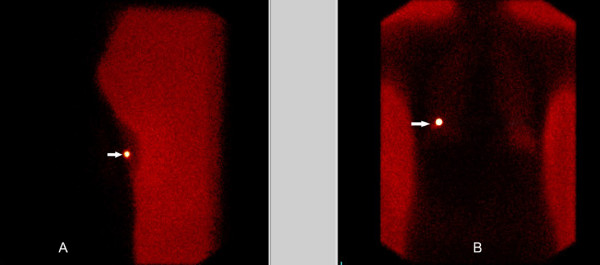
Lateral (A) and front (B) view planar scintigraphic images to check the radiographic correlation of a breast lesion (arrow).

One hundred and twenty patients with suspicious breast opacity or microcalcification cluster requiring diagnostic excision were randomized and submitted to guided surgical biopsy. WL was performed in 59 patients (49.2%) with standard techniques [2]. For ROLL (61 patients), 0.15 mCi (5.55 MBq) of ^99m^Tc-labeled macro albumin aggregate in 0.2 mL of saline was used. On the day of surgery, this solution was injected into the non-palpable lesion under mammography or ultrasound guidance. In addition, 0.1 mL of water-soluble non-ionic iodinated contrast medium was administered to check the exact position of the radiotracer at the time of injection. One hour later, the patient was submitted to front and lateral view planar scintigraphic images using a ^99m^TcO_4 _flood to check the radiographic correlation (Figure 1). The patient was taken to the operating room for excision of the lesion. Localization of the area of highest radioactivity was performed with a hand-held gamma probe (Navigator GPS™ – United States Surgical/Tyco Healthcare) to choose the most cosmetically acceptable site to incise. The specimen was excised after locating the highest radioactivity point and this hot spot was removed. It was located in the center of the specimen with a resection margin, with no excessive removal of normal breast parenchyma. The parenchyma bed was verified with the probe to rule out residual areas of high radioactivity. During surgery, a radiological study was performed to confirm total resection of cases previously demarcated by mammography. The use of local anesthesia for the ROLL procedure was proposed, considering that, contrary to the WL method, the wire is not maintained in the breast during the procedure. In all ROLL cases, local anesthesia (mean of 16 mL/patient of lidocaine with epinephrine-1:200000) was used in the skin and breast parenchyma close to the lesion. The hospital stay considered the period (in hours) between the beginning of the surgery and the discharge from the hospital. The procedure time was considered as the mean time of surgery in minutes. The patients were requested to score the cosmetic appearance of their breast as excellent, good, or poor in the first month after surgery. In addition, a numerical rating scale was used to measure pain on the firs postoperative day, considering a variation between 0 (no pain) and 10 (worst pain) [10,11]. Different statistical tests were used, when appropriate, in order to test the significance between the two groups, considering a P value < 0.05 as statistically significant. Clearance margin was considered as ≥ 10 mm for invasive cancer, ≥ 5 mm for ductal carcinoma *in situ*, and ≥ 1 mm for benign disease. All specimens were included with transversal serial cuts, with the margin size defined as the distance between the lesion and closest margin. Patients were subsequently treated according to the definitive histology result. This study was performed as per a protocol approved by the Ethical Review Board of Chapeco University.

## Results

Fifty-nine patients were randomized to WL and 61 to ROLL. The mean age of the two groups was 51.3 and 49.6 years, respectively. All procedures (in both groups) were done with diagnostic intention. The clinical and radiological characteristics of the two groups are summarized in Tables 1 and 2. There were no significant differences in the lesion sites and in the auxiliary localization technique in both groups. The ROLL technique did not increase the number of aesthetic incisions. The hospital stay was significantly longer in the WL group due to the use of general anesthesia (mean of 19.82 h vs. 2.04 h). This difference was statistically significant, but the result presents the bias that all the patients submitted to the WL technique needed a period of recovery from the anesthetic, i.e., a longer hospital stay. Procedure time was significantly shorter in the ROLL group (37.2 min vs. 26.06 min, respectively). The mean volume of the excised specimen was significantly smaller in the ROLL group than in WL group (8.70 cm^3 ^vs. 23.15 cm^3^, respectively). There was a difference in the margin status of the surgical specimens (P < 0.05), but these findings were not significant when considering only malignant lesions (Table 2). Postoperative wound infections between the groups were not significant. The mean of the numerical rating pain scale was different in both groups (2.20 for WL vs 1.62 for ROLL), and according to patients' opinions, there were better cosmetic outcomes with the ROLL technique (Table 3). In two cases of WL, there was wire dislodgement and the procedure needed repositioning.

**Table 1:** Clinical and radiological characteristics of WL and ROLL groups.

	WL	ROLL	P
Number of patients	59	61	
Mean of age (yr)	49.9 (35–77)	50.7 (32–76)	^£ ^0.540
Micro-calcifications	40	49	
Micro-Calcifications + Stromal deformity	2	3	
Stromal deformity	3	0	
Nodule	14	9	^£ ^0.080
Right Breast	17	20	
Left Breast	42	41	^£^0.351
SL Quadrant	22	27	
SM Quadrant	21	19	
IM Quadrant	6	4	
IL Quadrant	8	10	
Central	2	1	^£^0.948
Estereotaxy Localization	31	27	
US Localization	28	24	^£^0.130

^£ ^Chi-Square Test with Yates' correction

**Table 2:** Comparison of surgical and pathological features of WL and ROLL groups.

	**WL**	**ROLL**	**P**
Local of cutaneous incision			
Peri-areolar	21	31	
Others	38	30	^§ ^0.022
Histopathological Diagnosis			
Fibrocystic changes	25	34	
Fibroadenoma	12	14	
Invasive carcinoma	6	2	
Carcinoma *in situ*	10	8	
Others	6	3	^£^0.047
Size of specimen (mean in cm^3^)			
All lesions	23.15	9.70	^¥^0.001
Benign lesion	14.80	8.70	^¥^0.002
Invasive and not invasive carcinomas	20.30	9.45	^¥^0.170
Period of hospital stay (mean in hours)	18.7	3.06	^¥^<0.001
Anesthesia	General	Local	
Time of procedure (mean – min)	37.2	26.06	^¥^0.719
Margins of all lesions			
Clear	51	57	
Involved	8	4	^£^0.010
Carcinoma Margins			
Clear	14	9	
Involved	2	1	^#^0.670
Postoperative wound infection	1	2	^£^0.270

^§ ^Chi Square Test. ^£ ^Chi-Square Test with Yates' correction. ^¥ ^T-test. ^# ^Fisher exact

**Table 3:** Dependent variables on the patient opinion.

Techniques	WL	ROLL	P
Cosmetic outcome			
Excellent	49	57	
Good	10	04	
Poor	-	-	^§ ^<0.001
Pain (mean of numerical scale)	2.20	1.62	^¥ ^0.021

^§ ^Chi Square Test. ^¥ ^T – test

## Discussion

Nowadays, the diagnosis of subclinical breast lesions is very common due to easy access to standard mammography in most places. Many techniques, such as core biopsy, fine needle aspiration, and mammotomy are used for the histological study of clinically occult breast lesions. Sometimes it is necessary to excise all occult lesions in order to choose the adequate treatment. WL is a method used in many places as standard preoperative localization of non-palpable lesions. However, the problems reported with this technique are well known: wire transection, difficulties in wire repositioning in dense or fatty breasts, dislodgement, interference with the surgical approach, and patient discomfort during wire positioning and during patient transportation from the radiological center to the operating room [2,9,12].

Since 1996, when the first paper presented the advantages of ROLL, other authors have reported the same findings and have documented some characteristics of this technique: it is a radiologically and surgically easier procedure to perform, and the lesion can be identified in three dimensions affording greater flexibility in making a cosmetic incision [13]. ROLL is also appropriate for combination with sentinel lymph node mapping in which the occult breast cancer and sentinel lymph node can be excised in the same procedure [9,14,15]. Until now, the methodology for evaluation of postoperative pain has not mentioned the ROLL procedure, despite some works reporting postoperative pain when the WL is carried out [6,8,14,16]. The evaluation of pain on the first postoperative day and of the cosmetic outcomes was used as a parameter for comparing patients' opinions about both procedures, and there was a difference between the two groups. This is due to a better choice of an incision site (radioguided) and the fact that the size of the ROLL specimen is smaller. Hospital stay was shorter in the ROLL group due to the ambulatory characteristics of this procedure. Moreover, with the WL procedure, the patient needed a time to recover from general anesthesia. The failure rate of the wire guided technique (*i.e*. incomplete cancer resection) has been reported in the range of 40–50% [17]. The duration of the procedure was shorter in the ROLL procedure, which could be explained by better radioguided planning of the method; however, there were no significant findings (p > 0.05). The specimen size (mean in cm^3^) was smaller in patients submitted to ROLL and there were more cases with compromised margins with the WL procedure (p < 0.05), which again reflects better planning to include all lesions at the same time, and the specimen excised is the smallest possible. This difference, however, could affect the results for rates of cases with involved margins due to the different criteria of histological categories. To date, there have been no descriptions of a comparison between the use of local anesthesia in the ROLL procedure and use of general anesthesia in WL, especially comparing them as to aesthetic results and pain measurement.

## Conclusion

ROLL can provide diagnosis or treatment of the breast lesion with a shorter hospital stay, shorter operative period, less breast tissue excised, and consequently, better aesthetic outcomes and fewer procedure-related symptoms. It can result in lower costs and a better acceptance on the part of patients.

## List of Abbreviations

ROLL: Radioguided Occult Lesion Localization; WL: Wire-guided Localization; US: Ultrassonography.

## Conflict of interests

The authors declare that they have no competing interests.

## Authors' contributions

MM carried out all surgeries, participated in the interpretation of the study and drafted the manuscript. JEW participated in the execution of all ROLLs procedures by US or estereotaxy. BB participated in the execution and in the interpretation of the study. RLS participated in the design of study and performed the statistical analysis. BG aided to write the manuscript. LMBF conceived of the study, and participated in its design and coordination. All authors read and approved the final manuscript.
